# Correlation between Microstructural Alteration, Mechanical Properties and Manufacturability after Cryogenic Treatment: A Review

**DOI:** 10.3390/ma12203302

**Published:** 2019-10-11

**Authors:** Abbas Razavykia, Cristiana Delprete, Paolo Baldissera

**Affiliations:** Department of Mechanical and Aerospace Engineering, Politecnico di Torino, 10138 Torino, Italy; abbas.razavykia@polito.it (A.R.); cristiana.delprete@polito.it (C.D.)

**Keywords:** cryogenic treatment, cryo-treatment, mechanical properties, microstructure, cryo-processing

## Abstract

Cryogenic treatment is a supplemental structural and mechanical properties refinement process to conventional heat treatment processes, quenching, and tempering. Cryogenic treatment encourages the improvement of material properties and durability by means of microstructural alteration comprising phase transfer, particle size, and distribution. These effects are almost permanent and irreversible; furthermore, cryogenic treatment is recognized as an eco-friendly, nontoxic, and nonexplosive process. In addition, to encourage the application of sustainable techniques in mechanical and manufacturing engineering and to improve productivity in current competitive markets, cryo-treatment can be considered as a promising process. However, while improvements in the properties of materials after cryogenic treatment are discussed by the majority of reported studies, the correlation between microstructural alteration and mechanical properties are unclear, and sometimes the conducted investigations are contradictory with each other. These contradictions provide different approaches to perform and combine cryogenic treatment with pre-and post-processing. The present literature survey, mainly focused on the last decade, is aimed to address the effects of cryogenic treatment on microstructural alteration and to correlate these changes with mechanical property variations as a consequence of cryo-processing. The conclusion of the current review discusses the development and outlines the trends for the future research in this field.

## 1. Introduction

Subzero treatment is a deep stress relieving technology that is associated with cooling components below room temperature. Cryogenic treatment (CT) or cryo-treatment is a subzero heat treatment which is widely used in the production of high precision mechanical parts and components. It provides a large number of applications ranging from industrial components to improvement of musical signal transmission [1,2]. It encourages microstructural refinement and material property improvements such as wear resistance, toughness, fracture resistance, hardness, thermal conductivity, dimensional stability, and chemical degradation [3,4,5,6,7,8]. Accurate control of process parameters such as cooling rate, soaking temperature and time, cooling fluid, and the whole procedure, promotes the acquisition of superior mechanical properties. In addition, to encourage the application of sustainable techniques in mechanical and manufacturing engineering, and to improve productivity in the current competitive markets, cryo-treatment can be considered as a promising process to improve product service life and reduce production costs in terms of tooling cost and process interruption [9,10,11]. CT has been classified into three groups: cold treatment, shallow cryogenic treatment (SCT), and deep cryogenic treatment (DCT). Cold treatment is comprised of exposing the ferrous material to be treated in the temperature range of 193–273 K to refine microstructure and achieve better static mechanical properties. In contrast to heat treating, cold treatment does not require precise control of temperature, and its success depends only on the attainment of the minimum low temperature and is not influenced by lower temperatures [12,13]. In SCT, the samples are placed in a freezer at temperature range of 113–193 K and are then exposed to room temperature. In DCT, the samples are slowly cooled to 77–113 K, held down at this temperature for a certain duration, and are then gradually tempered to room temperature [14,15]. The DCT process consists of three main phases: cooling, soaking time, and warming. Each phase has significant effects on the mechanical properties and microstructure alteration, especially soaking time [16,17]. Generally, DCT is followed by some pre-and post-processing treatments [2,14,18]. While numerous advantages of DCT have been discussed in previous studies and were summarized in a dated review by the authors [14], there is a need for investigating the cryogenic treatment in relation to pre-and post-treatment and their influences on microstructural alteration and mechanical properties. Besides, there are contradictions within the literature due to a lack of understanding of the fundamental metallurgical mechanisms of microstructural alteration and their correlation with the mechanical properties. Therefore, the current review survey is devoted to clarifying the effect of CT on microstructural changes and the correlation between these alterations and the mechanical properties, with a special focus on the knowledge deriving from more recent studies. This review is organized into three main sections: first, all the possible alterations induced by cryo-treatment on the microstructure will be discussed; second, the effects of these microstructural variations on the mechanical properties and their correlation will be addressed; finally, a summary of the conducted experimental investigations on cryo-treatment application in manufacturing engineering will be presented.

## 2. Effects of CT on Microstructure Alteration

There are imposed stresses whenever a material undergoes any manufacturing process which follows the mechanical component’s production. In addition, there are some stresses due to defects in the crystal structure of the material, of which vacancies, dislocations, and stacking faults are the most common [19]. The structural defects are proportional to the level of the imposed stresses when the materials undergo manufacturing processes and cyclic stresses while the materials are under service. Cyclic stresses encourage the defect’s migration and their accumulation within the matrix, while the further movement of defects requires more energy. The higher the level of stress, the greater the degree of the defect’s migration, which leads to an increase in the interatomic spacing. Consequently, crack nucleation starts and propagates as the distance between the atoms exceeds a critical distance.

**Figure 1:**
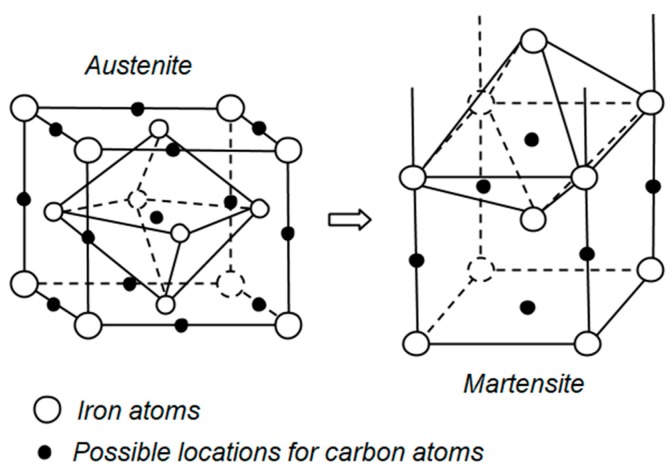
Transformation of soft retained austenite to relatively hard and stable martensite at lower temperature [30].

For different materials, distinctive alteration scenarios take place in the microstructure during CT [20]. The magnitude of these changes is affected by the treatment process parameters. In most steels, the transformation of retained austenite into martensite [21,22,23,24], fine carbide precipitation [25,26], and uniform distribution of secondary carbides [3,27], as well as interstitial carbon atoms segregation, are the common changes observed in the microstructure after CT execution in comparison to the conventional treatments (quenching and tempering) [28]. Applying the appropriate temperature and soaking time causes substantial transformation of the soft austenite with a face centered cubic (FCC) crystal structure into hard martensite with a body centered cubic (BCC) structure, as shown by Figure 1. In addition, metallurgical alteration of martensite at a lower temperature can be obtained [29,30]. These metallurgical changes in martensite are in the form of a reduction in brittleness and an increased resistance against plastic deformation [31]. The formation of fine and very small carbide particles dispersed within the martensite [25,32] provides more interfaces with the base martensite matrix. These interface increments provide more obstacles against dislocation movements and prevents the foreigner particles from penetrating into the matrix, thereby improving the abrasion wear resistance [33,34]. Due to differences in the crystal size of austenite and martensite, there will be imposed stresses where both coexist. CT, and especially DCT, eliminates these stresses by transforming austenite to martensite. Interstitial carbon atom segregation near dislocations is observed during CT, which acts as growing nuclei for the formation of fine carbide particles on subsequent tempering [35,36].

## 3. Effects of CT on Microstructure Variation and Hardness

**Figure 2:**
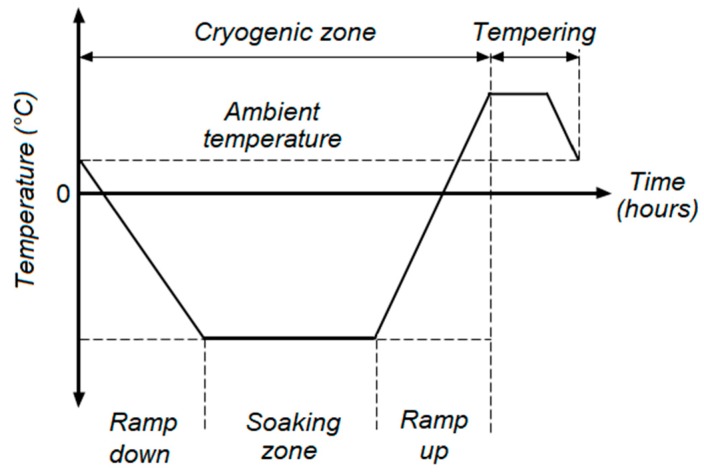
Deep cryogenic treatment (DCT) with single tempering.

There is a continual demand to improve mechanical properties of industrial components as their complexity grows and functionality requirements increase. Therefore, DCT can be recognized as an alternative process to obtain better mechanical properties with microstructural manipulation. Figure 2 exemplifies a general illustration of the DCT process. DCT improves hardness via two mechanisms, precipitation hardening and martensitic transformation. This section is aimed at providing an insight into the hardening mechanisms contributed by DCT.

**Figure 3:**
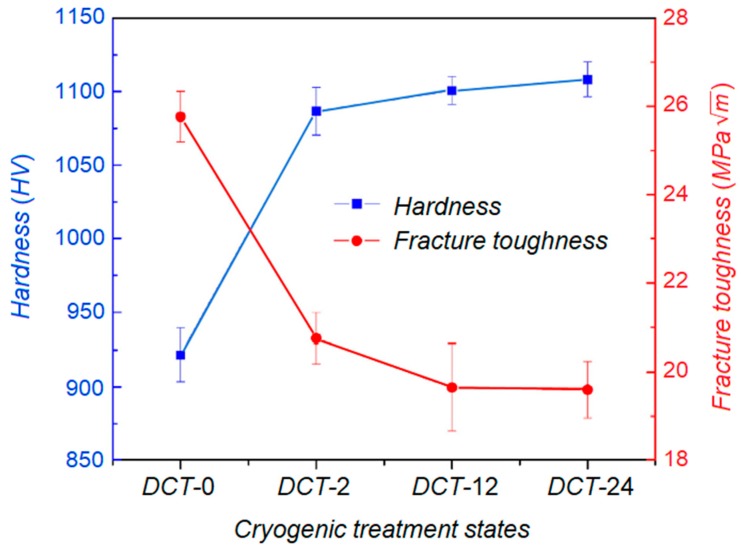
The hardness and fracture toughness of WC-Fe-Ni cemented carbides before and after DCT with different soaking time [38].

A reduction in the level of retained austenite, or even its elimination, can be substantially obtained if DCT is performed efficiently in a vacuum environment. Leskovšek and Ule carried out DCT on AISI M2 high-speed steel to reach a compromise between hardness and fracture toughness [37]. Furthermore, tool shape and dimensional stability have been examined. It was observed that the volume fraction of the retained austenite, and hardness, have a paramount impact on the fracture toughness. DCT promotes a higher rate of retained austenite transition into martensite which improves the hardness, while at the same time, worsens the fracture toughness. Figure 3 shows the effect of DCT on hardness and fracture toughness and their intercorrelation [38]. The combination of DCT and a vacuum environment encourages a higher austenite to martensite transformation and better dimensional stability. In contrast, shape distortion was detected due to the combined effects of transformational and thermal stresses.

Zhirafar et al. conducted an observation to evaluate the alteration of mechanical properties of AISI 4340 after DCT [39]. Higher hardness was detected and consequently, the fatigue limit was improved because of the austenite transformation into martensite. The interaction between hardness and fatigue limit is anticipated to be influenced due to the microstructural changes in steel, considering that the microstructural alteration in steel encourages the achievement of a linear trend between hardness and fatigue limit, even for higher values of hardness [40].

An experimental investigation has been conducted to trace a coherent picture of the effects of DCT on microstructure variation [41]. The complete transformation of austenite to martensite, coupled with a higher volume fraction of fine carbides in the martensite matrix, improved the hardness of En 31 steel as result of DCT and subsequent tempering treatment.

Harish et al. tried to understand the impact of DCT and SCT on En 31 bearing steel microstructural alterations [42]. Higher hardness was obtained through the CT in comparison to conventionally treated samples. Fractography, by means of optical microscopy (OM), provided evidence of equiaxed dimples and flat facets present in the SCT workpiece, and a wide size range of dimples and microcracks in the DCT specimen. Retained austenite and a fine distribution of medium size spheroidized carbide particles have been observed after DCT. Tempered martensite, some spherical carbide particles, and an amount of retained austenite were detected due to SCT execution after tempering. Some needle-like regions were observed in the DCT microstructure prior to tempering, which implies the presence of untampered martensite and the existence of some retained austenite with a lower volume fraction, in comparison to SCT and conventional heat treatment (CHT). Meanwhile, the existence of retained austenite in SCT and DCT structures proved that CT boosts the formation of martensite from retained austenite, but a complete transformation was not observed even after DCT and SCT.

An investigation has been conducted to study the impact of DCT on the microstructure and mechanical property alterations of cold work die steel (Cr8Mo2SiV) [43]. It was observed that the content of precipitation carbides is influenced by the soaking time. DCT results in a martensite and austenite lattice contraction with a homogeneous carbide distribution, and consequently, carbon atoms are forced to diffuse and make a new carbide nucleus. Regarding these alterations, the carbide precipitation content is dependent on the repeating time. Therefore, the volume fraction of the carbide precipitation is affected by both the soaking time and the repeating times. At the end of the process, the hardness of the DCT samples was found to be greater than those obtained by conventionally treated workpieces (quenching and tempering).

HS6-5-2 high speed steel microstructure alteration after DCT has been examined by means of transmission electron microscopy (TEM) and differential scanning calorimetry (DSC) [44]. Qualitatively, it was observed that the martensite obtained after quenching, DCT, and heating up to the ambient temperature was formed by a lamellar-lenticular structure, and internally twinned with a very high density of dislocations. Homogeneous distribution of spherical carbides within the martensite grains is responsible for the hardness improvement.

The effects of SCT and DCT on surface residual stresses, hardness, and impact toughness of 4140 steel have been compared [45]. Higher residual stresses were generated after DCT, in comparison to quenching and further SCT, due to a reduction in the density of lattice defects (dislocations) and thermodynamic instability of the martensite. These effects motivate the movement of carbon and alloying elements toward the defects, and this movement, as mentioned, acts as a basis to form fine carbides after stress relief. It was highlighted that the precipitation of carbides in tempered SCT and DCT samples is responsible for the residual stress relaxation. Meanwhile, the temperature reduction of CT encourages a higher transformation of austenite into martensite and at the same time greater compressive residual stress in the untempered DCT specimens. It is worth mentioning that in contrast to tensile stresses in CHT and SCT workpieces, compressive stresses were observed after DCT.

Mehtedi et al. examined the effects of DCT on treatment response, microstructural alteration, and their correlation with X30 CrMoN 15 1 steel hardness [46]. It was noted that DCT encourages the transformation of the retained austenite to martensite and a homogeneous decoration of the martensitic matrix by refined carbides particles, with a consequent improvement in hardness. It is worth mentioning that, generally, hardness variation is proportional to the austenitizing temperature.

Amini et al. examined the effects of soaking time in liquid nitrogen on the microstructural alteration, carbide distribution, and volume fraction, as well as hardness of 1.2080 tool steel [34]. In all the different soaking times, the elimination of retained austenite, an increment of carbide particles density with homogenous distribution, and a uniform size were observed. Nevertheless, the magnitude of these alterations was affected by the soaking temperature. Hardness improvement was obtained by DCT, but further changes were not noted after a certain holding duration (36 h).

Finite element analysis considering the transformation kinetics has been conducted to simulate DCT and was experimentally validated [47]. Comparison of the experimental data with the numerical method reveals that multiphysical field coupling simulation is an effective and accurate approach to evaluate the cooling behavior of DCT. It was observed that the amount of retained austenite significantly decreased, and as a result, hardness was improved.

Comparative investigation has been conducted to contrast CHT, SCT, and DCT effects on AISI M35 HSS microstructural variations [48]. A higher reduction in the volume fraction of retained austenite was obtained by DCT, followed by SCT. A larger amount of fine precipitations of carbides was observed after DCT. The combination of retained austenite reduction and the distribution of the carbide precipitation resulted in a hardness improvement.

Akhbarizadeh and Javadpour investigated the effect of the as-quenched vacancies on the carbides formation in the microstructure of 1.2080 tool steel after DCT [49]. The effect of the vacancies as potential sites for carbon atoms’ jumping during DCT was clarified by using an electric current. It has been noticed that the as-quenched vacancies play a significant role in the carbide formation during DCT by providing appropriate sites for the carbon atoms’ jumping, which results in a hardness improvement. The authors concluded that these carbon atoms provide some appropriate places for carbide nucleation during the tempering.

**Figure 4:**
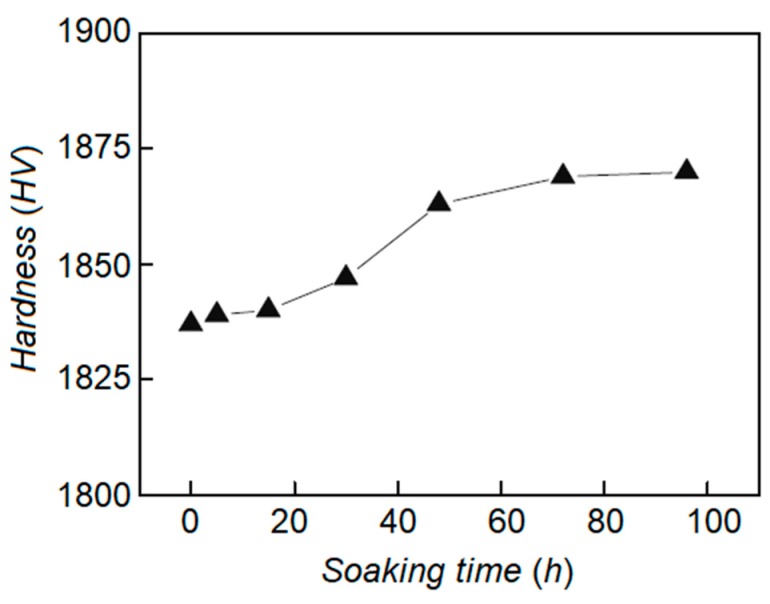
The effect of soaking time on hardness variation of the ultrafine-grained WC–12Co cemented carbide [51].

A comprehensive investigation has been conducted to study the microstructural and mechanical properties alteration of ultrafine-grained tungsten carbide–cobalt WC–12Co cemented carbide subjected to DCT. The phase transformation of the binder phase Co and the hardness over a wide range of temperatures was studied using thermal analysis and selective electrolytic corrosion technology as shown in Figure 4. In contrast to previous studies [50], the precipitated tiny second-phase particles could not be observed in the Co binder phase due to magnification limits associated with scanning electron microscopy (SEM). X-ray diffraction (XRD) analysis revealed that DCT promotes transformation of ɛ-Co, and there is no influence on the crystal structure of tungsten carbide (WC) particles and consequently, this improves the hardness and bending strength of the cemented carbides [51].

A comparative investigation between CHT, SCT, and DCT was conducted to evaluate microstructural variation and hardness of AISI 440C bearing steel [52]. A higher rate of retained austenite transformation into martensite was achieved by DCT and a lower volume fraction of martensite was traced by CHT, while DCT samples showed higher hardness.

Xie et al. investigated the influence of DCT on the microstructure and mechanical properties of WC−11Co cemented carbides with different carbon contents [53]. DCT has a nonsignificant effect on phase composition. The amount of η-phase was increased as compared to untreated samples. It was observed that DCT refines the WC grains into triangular prisms with rounded edges, without size alteration, through the spheroidization process. In addition, the phase transformation of the Co phase from α-Co (FCC crystal structure) to ε-Co (HCP crystal structure) was observed after DCT, with reduction of W solubility in the binder (Co). It was highlighted that DCT improved the hardness and bending strength of the alloys, but there was no remarkable impact on the density and cobalt magnetic performance.

Yuan et al. studied the microstructural alteration and mechanical properties of commercially pure zirconium after DCT execution [54]. The DCT reoriented grain is much closer to the (0 0 0 1) basal plane and encouraged a higher grain boundary misorientation and dislocation density. Therefore, the higher fraction of grain boundary misorientation provides more obstacles against the dislocation movement, increasing the material resistance against plastic deformation and improving the hardness. Furthermore, the basal planes are associated with a higher hardness in comparison to the prism planes.

Pérez and Belzunce conducted an observation to examine the influences of DCT on the mechanical properties of H3 tool steel [55]. DCT lessens the retained austenite content in H13 steel, but there is a minimum innate content which cannot be transformed by heat treatment. The H3 steel hardness decreased, as the carbide precipitation and carbon content of the martensite reduced.

**Figure 5:**
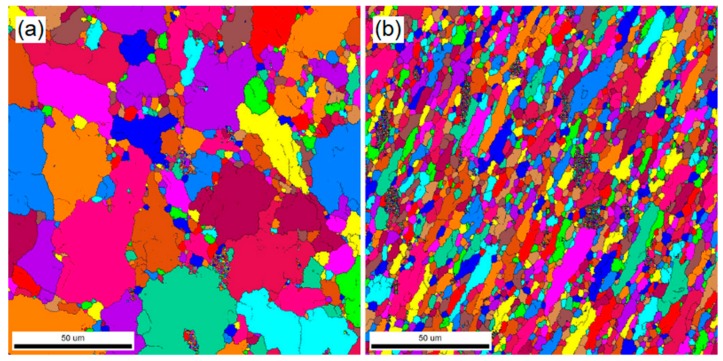
Electron back scattered diffraction (EBSD) micrographs of (**a**) the base and (**b**) the shallow cryogenic treatment (SCT) sample [56].

Mohan et al. examined the SCT impact on the microstructure and mechanical properties of Al7075-T6 [56]. Static mechanical properties such as hardness, yield strength, and ultimate tensile strength were improved. Precipitation, better distribution of second-phase particles, and higher dislocation density were observed using electron back scattered diffraction (EBSD) after DCT treatment in comparison to untreated specimens, as illustrated in Figure 5. It was highlighted that the hardness and stiffness improvements are the consequence of precipitation hardening and high dislocation density. Fatigue limit has been improved due to striations becoming denser in the cryo-treated alloy.

A set of subzero treatments (SZTs) were conducted on aluminum 2024, to study microstructure, hardness, and tensile and fatigue strength [57]. SZTs were performed at −60 °C (held for 10 h) and at −196 °C (held for 4 h). As the grain size and formed nanoparticles in the microstructure are refined by applying SZTs, elongation, yield strength, hardness, and tensile strength were improved. In contrast, due to microcrack formation in the SZTs, the fatigue limit was reduced under both treatment protocols.

An investigation was designed to examine the effects of the amount of particle refinement and different subzero and ageing processes on the hardness of Al7075 [58]. A smaller atomic distance, refined particle shape, and uniform microstructure were observed after treatments. In addition, the refined microstructure encourages higher friction against dislocation movement, and thus higher hardness.

The current literature survey has a broad consensus about the fact that CT provokes the microstructure alteration and consequent changes in the mechanical properties as summarized by Table 1. In the case of steels, performing a complete treatment process, comprising of austenitization, quenching, cryo-treatment, and tempering, would promote a more refined microstructure and improved mechanical properties. Austenitizing and quenching transform some austenite and primary carbides into martensitic phases. Tempering encourages transition carbides by means of the transformation of supersaturated carbon to form carbides. This contribution relieves microstresses in the martensitic structure and prevents crack nucleation. The effects of CT on steels can be attributed to some important changes in the microstructure that affect the hardness:

- Transformation of retained austenite to martensite can be boosted under vacuum conditions. However, complete transformation cannot be achieved after cryo-treatment as some untempered martensite, characterized by needle-like regions and some volume fraction of retained austenite, has been observed. In this stage, tempering would assist in ameliorating the transformation of retained austenite to martensite and refining the martensitic structure to obtain better hardness;
- Higher volume fraction of fine carbides within the martensite matrix reinforces the martensitic structure and improves hardness;
- Homogeneous distribution of carbides, and a good decoration of the martensite matrix with small size carbide particles, provides more resistance against the dislocation migration within the matrix and plastic deformation;
- Martensite and austenite lattice contractions, along with the uniform distribution of refined carbide particles, encourages carbon atoms diffusion and new carbide nucleation, which results in a higher volume fraction of carbides, especially during tempering, and consequently improves the material hardness;
- Soaking time and temperature, as well as tempering rate, have a prime importance in improving hardness.

Cemented carbides after CT undergo some changes within the microstructure, which bring better mechanical properties. Transformation of the binder phase Co from α-Co to ɛ-Co, an increment of the η-phase volume fraction, and a reshaping of the WC grains without size reduction, are the effects of DCT which improves the bending strength and hardness.

In the case of titanium alloys, DCT imposes an alteration in the volume fraction, size and morphology of the α and β phases and improves hardness.

## 4. Effects of CT on Microstructure Alteration and Wear Resistance

Enhancement of the wear resistance of loaded components with relative motion is a promising approach to improve efficiency and durability for a wide range of machine elements in tribo-systems. CT would be a reliable option to improve wear resistance of components via material microstructure modifications. Therefore, this section highlights the achievements of previous studies to share the technical synthesis with an emphasis on correlation between microstructural variations and wear behavior.

Thakur et al. compared the impacts of three different post-treatments on tungsten carbide–cobalt inserts’ microhardness and microstructural alteration [59]. The effects of controlled cryo-treatment, heating and forced air cooling, and heating and quenching of WC–Co in an oil bath have been investigated. A slight increment of microhardness was obtained by CT and the higher microhardness was measured by the other treatments. A wear resistance improvement after controlled CT was detected due to the densification of the cobalt metal binder which holds the carbide particles firmly.

The effect of austenitizing time in DCT on the wear resistance of D6 tool steel was measured using a pin-on-disk wear test [60]. By increasing the austenitization time from 10 to 50 min, grain size experienced an increment of 122%. It was observed that the diffraction intensities of the martensite increased, while the volume fraction of austenite decreased, which implies that the prior austenite grain size does not affect the retained austenite volume fraction after DCT. After conducting hardness tests, it was concluded that the austenitizing time plays an important role on microstructure homogenization, which encourages higher wear resistance.

Dhokey and Nirbhavne conducted a comparative study to evaluate conventional quenching, tempering, and intermediated CT, and their effects on the wear resistance of D3 tool steel [61]. A huge segregation of carbides of massive size was observed after conventional quenching, but tempering encouraged the reduction in carbide size, and CT resulted in a higher volume fraction of fine carbides and their nucleation during ramp up. Development of fine carbides after CT improves the wear resistance against sliding wear, which could be attributed to the formation of nanosized η-carbides.

A set of treatments were proposed to study the effect of microstructural alteration on wear behavior of D6 tool steel [62]. SCT at −63 °C and DCT at −185 °C were carried out to examine the effects of treatment types, soaking time, and stabilization. Wear tests were conducted applying pin-on-disk wear tests using different loads and sliding speeds. It was observed that CT improved the wear resistance by means of reducing the amount of retained austenite. However, DCT became more efficient in comparison to SCT, as it encouraged a more homogenized carbide distribution and elimination of the retained austenite. The longer the treatment time, the higher the wear resistance and hardness as more retained austenite was transformed into martensite.

**Figure 6:**
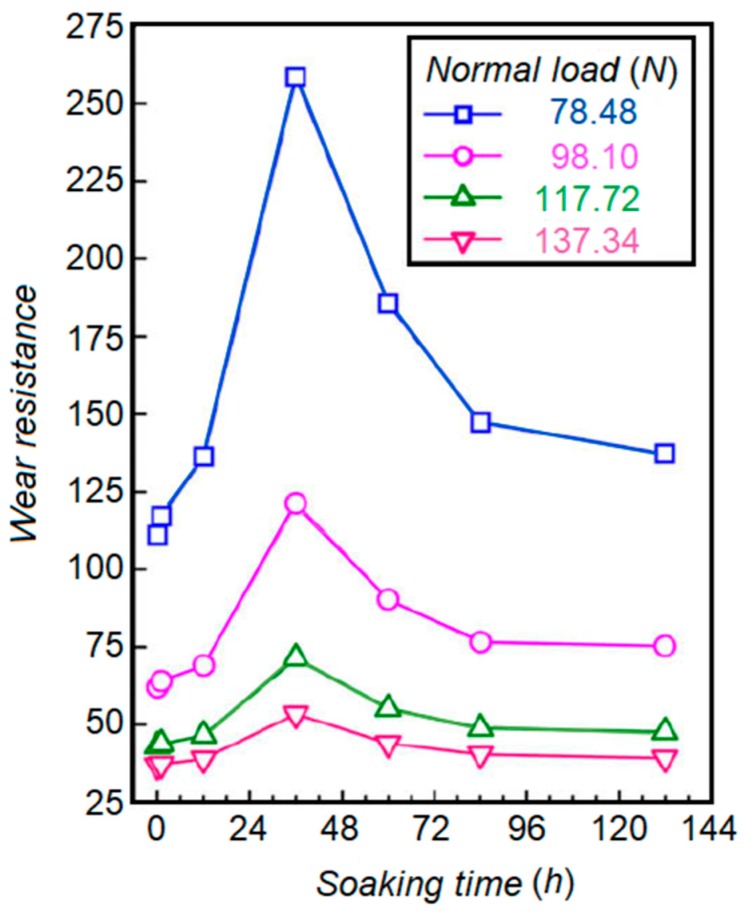
Influence of soaking time on the wear resistance of cryo-treated D2 steel specimens [63].

Das et al. tried to correlate the soaking time and wear behavior of AISI D2 to find the optimum soaking duration in the range of 0 to 132 h, and a cryogenic temperature of 77 K [63]. Figure 6 demonstrates the effect of soaking temperature on wear resistance of AISI D2 specimens subjected to different loads. The results provide evidence that there is a strong correlation between soaking time and the precipitation behavior of secondary carbides. Wear behavior of the cryo-treated AISI D2 samples is affected by the volume fraction of secondary carbide particles within the matrix. The optimum time of around 36 h for the soaking zone of the considered material was recommended.

Straffelini et al. optimized the wear resistance of stamping tools applicable to the automotive industry [64]. The effects of three treatments were examined: coating by thin ceramic film (AlCrN) using physical vapor deposition (PVD), a tool produced by hard metal, and two DCT treated HSS (high-speed steel). DCT improves the wear resistance of the tool as a result of the precipitation of ultrafine carbide particles.

The influence of DCT parameters, soaking time, and temperature on the tribological performance of powder–metallurgy (PM) high-speed steel were investigated by means of abrasive wear resistance and resistance to galling under dry sliding conditions [65]. It was observed that a higher austenitizing temperature leads to a smaller amount of undissolved eutectic carbides of small size. The longer treatment time results in a fine microstructure. It was concluded that the austenitizing temperature is a more significant player in comparison to the soaking time.

**Figure 7:**
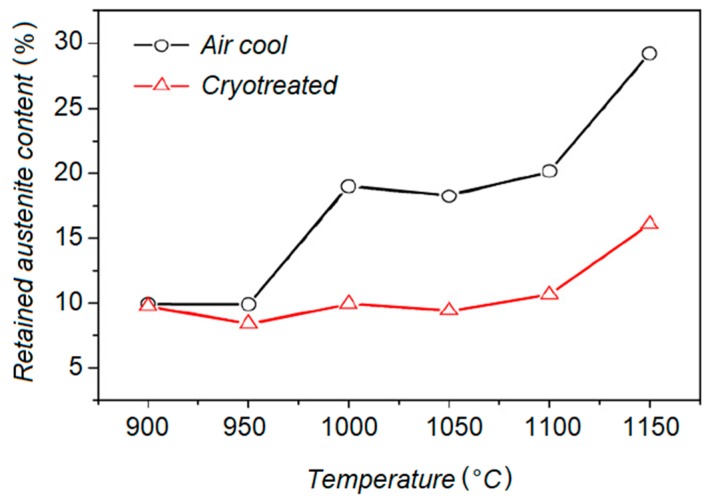
The content of retained austenite at different heat-treated states (heated for 0.5 h) [66].

Wang et al. studied the effect of DCT on the microstructure and abrasion resistance of a high chromium cast iron [66]. It was observed that the volume fraction of secondary carbides increased after the destabilization treatment combined with DCT. Cryo-treatment resulted in the transformation of the abundant retained austenite into martensite and finer secondary carbide precipitated formation, which are the main contributors to wear resistance improvement. Figure 7 compares the volume fraction of retained austenite after air cooling and cryo-treatment following the destabilization treatment. Therefore, a higher volume fraction of carbide content provides more interface with the matrix, which promotes obstacles against dislocations and enhances the wear resistance.

The effects of DCT parameters, including austenitizing temperature, ramp down, soaking time, ramp up, and tempering temperature, on AISI D2 wear behavior have been examined using the Taguchi method [67]. It was observed that complete transformation of retained austenite to martensite cannot be achieved during ramp down, but the greatest transformation was achieved during soaking time and was affected by soaking temperature.

**Figure 8:**
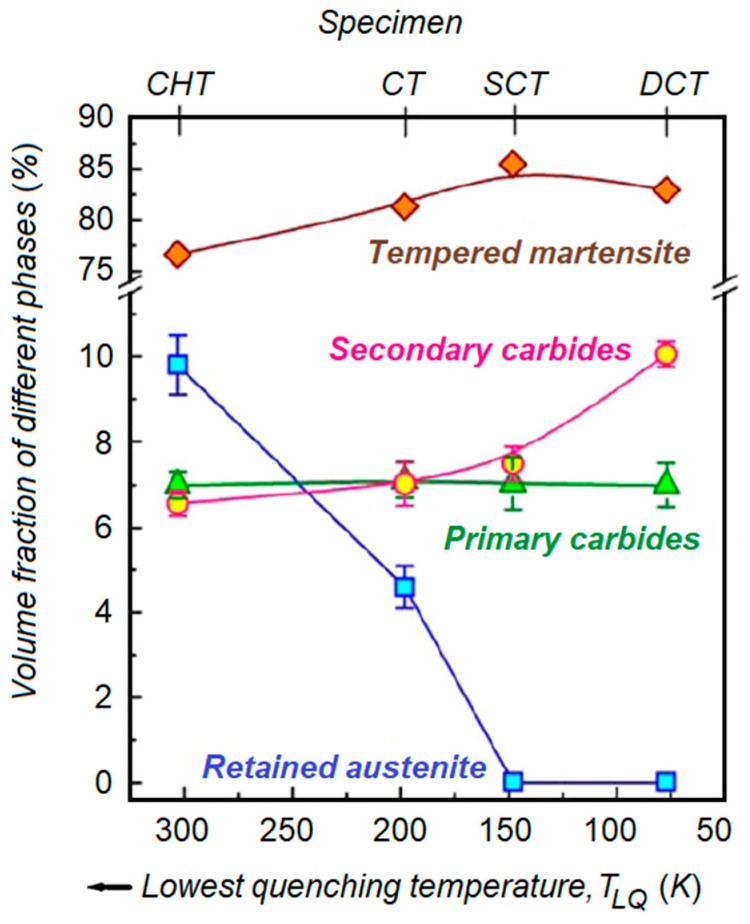
Variation of number of different phases with lowest quenching temperature (TLQ) [68].

Das et al. studied the advantages of cooling temperature during subzero treatment on AISI D2 steel wear resistance [12,68]. Wear behavior was evaluated by estimating specific wear rates and conducting detailed characterizations of the worn surfaces, wear debris, and subsurfaces using SEM coupled with X-ray. Figure 8 compares the effects of treatment processes, CHT, and subzero treatments (CT, SCT, and DCT) on the volume fraction of different phases within the matrix. It was observed that the lower the temperature of subzero treatment, the higher the wear resistance. RA reduction in retained austenite volume fraction and the simultaneous increment in the amount of secondary carbide particles at lower temperature improved the wear resistance.

Cajner et al. studied the influence of DCT on standard PM S390 MC high speed steels [69]. The effects of DCT on impact and fracture toughness, erosion wear resistance, and microstructure were examined. DCT brought some advantages, such as remarkable improvement of wear resistance due to the presence of η-carbides, however a significant change in toughness was not recorded.

The effects of DCT on microstructure, creep, and wear resistance of AZ91 magnesium alloy have been examined [70]. Alteration of β precipitates distribution, the coexistence of dissolved tiny laminar β particles, and coarse divorced eutectic β phase, were observed as results of DCT. It was highlighted that the creep behavior of the alloy, which is affected by the stability of the near grain boundary microstructure, was improved. Wear resistance was enhanced after DCT as consequences of the internal microstructure stabilization and the formation of a new morphology of β particles.

Sogalad and Udupa conducted an investigation to evaluate the DCT impact on the load bearing capacity of fitted pairs (En8 steel) interference [71]. Different soaking times were applied to the pins sunk in liquid nitrogen and ice. In addition, the bushes were heated to be assembled with the pin without external pressure. Stronger joints were obtained after DCT due to an improvement of the hardness and wear resistance as a consequence of the austenitic transformation to martensite. The improved wear resistance of the pins is attributed to fine carbide formation with a tight lattice structure due to DCT impact. The load carrying capacity of the bearing was improved after cryo-treatment due to a transformation of the retained austenite to freshly formed martensite and the distribution of η-carbides, which fill the microvoids present in the matrix, making it much denser and more uniform.

Akhbarizadeh et al. applied an external magnetic field during DCT to evaluate wear behavior of 1.2080 tool steel [72]. It was observed that DCT reduces the volume fraction of retained austenite and encourages the uniform distribution of carbide particles within the matrix, and consequently, enhances the wear resistance. Surprisingly, the magnetic fields had the reverse effects and reduced the number of carbides, with a nonuniform distribution, which worsened the wear resistance.

**Figure 9:**
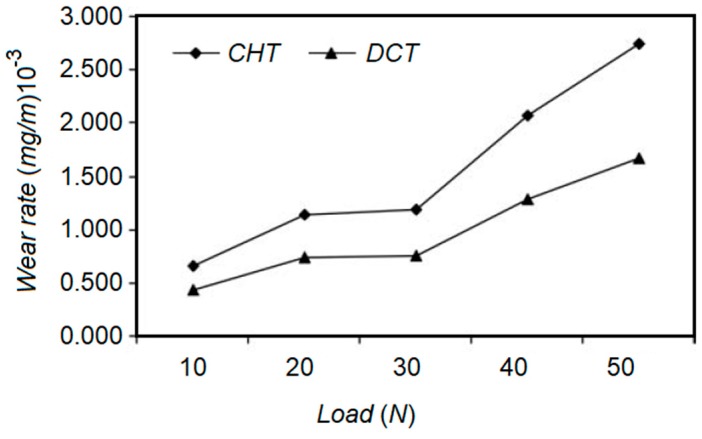
Wear rate of 100Cr6 bearing steel at 5 Hz frequency for the conventional heat treatment (CHT) and DCT [73].

Siva et al. investigated the effects of DCT execution on the wear resistance of 100Cr6 bearing steel [73]. Significant improvement of wear resistance after DCT was obtained in comparison to conventionally treated specimens (Figure 9) which was attributed to the alteration of the retained austenite into martensite and the precipitation and distribution of the carbides within the microstructure. DSC revealed that the increment of martensite destabilization by triggering carbon clusterization and carbide precipitation resulted in an improvement of wear resistance and hardness.

Xu et al. evaluated high performance tool steels’ (AISI H13) microstructural alteration prior to and after CT using XRD and synchrotron microdiffraction [74]. The execution of DCT results in diffusion of excess carbon out of the martensite phase and martensitic unit cell shrinkage. Besides these changes, cryogenically treated samples received homogeneous martensite structure. It has been highlighted that a considerable degree of disorder, resulting from rapid cryogenic cooling, encourages microstresses discharge during tempering, which improves wear resistance.

Arockia, Jaswin, and Mohan carried out an observation to optimize the CT with the aim of improving wear resistance, hardness, and tensile strength of E52 valve steel by applying the Grey–Taguchi method [75]. Different combinations of cooling rate, soaking temperature, soaking period, and tempering temperature were considered to execute the DCT. Significant alteration of the retained austenite to martensite and an increment of the fine secondary carbide precipitation were obtained in optimized DCT specimens. Therefore, an improvement of hardness and wear resistance was achieved as a consequence of the aforementioned effects.

Li et al. conducted a set of experimental observations to study the tribological performance of tool steel subjected to DCT [76]. It was highlighted that the segregation of carbon atoms to nearby dislocations, and the interaction produced between themselves and the dislocations, encouraged the formation of carbon clusters. These carbon clusters act as nuclei to form carbide particles during subsequent tempering, and consequently improve wear behavior.

The effects of DCT on the microstructural alteration and wear behavior (abrasion characteristics) of H13 tool steel have been observed [77]. It was highlighted that the lower the temperature, the higher the amount of transformed retained austenite to martensite, resulting in smaller and more uniform distributions of martensite laths. Furthermore, DCT promotes the precipitation of more homogeneous and very fine carbide particles. The joint effects of these microstructural variations improved the wear properties of the H13 tool steel.

Senthilkumar and Rajendran examined the effects of DCT on the wear resistance of En19 steel [78]. Three different types of heat treatment were performed. The influences of DCT, SCT, and CHT were investigated through dry sliding wear testing. X-ray observation revealed that the precipitation of fine carbides and the transformation of retained austenite into martensite, during DCT and SCT, improved the wear resistance in comparison to CHT. It was highlighted that a higher wear resistance and lower friction coefficient have been obtained by DCT.

Jaswin et al. evaluated the effects of SCT and DCT on the microstructure and wear resistance of X45Cr9Si3 and X53Cr22Mn9Ni4N valve steels [79]. The alterations were compared to conventionally heat-treated specimens. SEM analysis reveals that the full elimination of the retained austenite is not achievable even after performing SCT and DCT, but a reduction in the retained austenite was observed as a consequence of CTs. It was highlighted that the formation of fine carbides dispersed in the tempered martensite structure is the main contributor to improved wear resistance, followed by the retained austenite transformation.

Li et al. carried out a set of experimental trials to evaluate DCT effects on internal friction behaviors in the process of tempering [80]. Wear resistance improvement was observed due to greater carbide precipitation after DCT by means of applying internal friction. This is because as the carbon atoms move towards the dislocations, strong interaction is generated between interstitial carbon atoms and among them with a time dependent strain field of dislocations. The generation of carbon atom clusters adjacent to the dislocations under DCT, inspires carbide formation which subsequently results in wear resistance improvement.

Amini et al. investigated the effects of stabilization, tempering, and DCT on the mechanical properties of 80CrMo12 5 tool steel [81]. Elimination of the retained austenite and a higher amount and finer distribution of carbide, as a consequence of DCT, dramatically improved the wear resistance. Ultimate tensile strength increased and a reduction in specimen toughness were also observed. It was concluded that DCT treatment should be done immediately after quenching to obtain the highest wear resistance and hardness.

**Figure 10:**
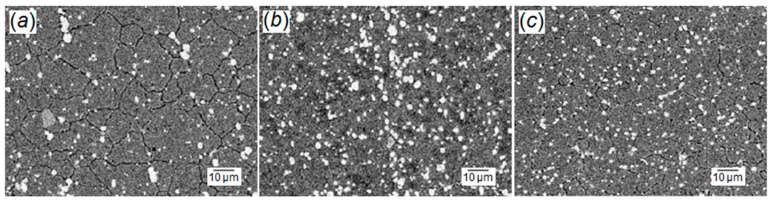
SEM image used for quantitative determination of the size and volume fraction of carbides for (**a**) CHT, (**b**) SCT, and (**c**) DCT [82].

Gill et al. conducted a set of trials to examine the effects of DCT on the mechanical and microstructural alteration of AISI M2 HSS [82]. It was proved that complete transformation of austenite into martensite cannot be achieved by DCT, however some advantages, such as higher precipitation of small carbides, an increment of their volume fraction, and uniform distribution of the carbides, have been obtained after DCT followed by SCT, as shown in Figure 10. Wear resistance improvement was observed due to the aforementioned DCT and SCT effects.

Sri Siva et al. applied the Taguchi method and Gray relational analysis to optimize the DCT processes to be executed on 100Cr6 bearing steel [83]. Dimensional stability, wear resistance, and hardness were selected as the indicators to examine the effect of cooling rate, soaking temperature, and duration, as well as tempering temperature, to be proposed for optimization. It was highlighted that the precipitation of the fine carbides and the transformation of the retained austenite to martensite are the main contributors to the improvement of hardness, wear resistance, and dimensional stability.

Isothermal martensitic transformation at temperatures below −100 °C during DCT was studied as a physical mechanism that has a significant contribution to mechanical properties improvement [84]. Steel X153CrMoV12 was selected on which to conduct the observations. Findings revealed that the joint effects of low temperature martensitic transformation and plastic deformation play a remarkable role in the subsequent carbide precipitation, which improved the mechanical properties such as abrasive wear resistance and hardness. Low-temperature isothermal martensitic transformation, as a result of unbalanced volumes of the martensite and retained austenite, leads to plastic deformation which encourages the refining of primary and secondary carbides and consequently, improves hardness.

A study was designed to determine the effects of influential variables on the correlation between treatment parameters and mechanical properties of AISI D2 steel subjected to distinct categories of SZTs viz. cold, SCT, and DCT [85]. The effects of the treatment process were examined by means of the number of different phases and stereological parameters of the secondary carbide (SC) particles. It was highlighted that a reduction in the retained austenite, and at the same time, an increment of SCs by subzero treatment, resulted in an improvement of bulk hardness and microhardness with a minor fracture toughness loss, and a remarkable improvement in wear resistance.

The effects of DCT on D2 tool steel wear resistance have been studied by means of pin-on-disk tests using steel and tungsten carbide pins [86]. Microstructural alteration and wear mechanism were evaluated using SEM and XRD. Wear behavior was improved due to the formation of fine carbides with the size varying from micron to nanoscale. In addition, a higher amount of carbide content and homogeneous carbide distribution were observed after DCT treatment. Adhesive wear was the predominant mechanism under different observational set-ups (load, sliding speed, and pin material).

Das and Ray studied the mechanism of wear resistance improvement by applying DCT [87]. It was highlighted that DCT eliminates retained austenite and encourages the formation of secondary carbides and consequently enhanced the wear resistance of AISI D2 steel.

Podgornik et al. reported that DCT improved the abrasive wear resistance and better galling properties of powder–metallurgy (PM) high-speed steel, as a result of a finer needle-like martensitic microstructure formation which provides a lower average friction coefficient, while applying longer soaking time [88].

The effects of different soaking and tempering times during DCT of 45WCrV7 tool steel have been studied [89]. Maximum hardness was obtained by increasing either the soaking time or tempering time, as these two encouraged the higher transformation rate of retained austenite into martensite, in addition to a larger volume fraction of carbides with a homogenous distribution and uniform size. It was proved by another investigation that the joint effect of soaking and tempering times is the most influential parameter to enhance hardness and consequently, wear resistance [90].

An AZ91 magnesium alloy was subjected to DCT and the microstructural alteration was examined using OM and SEM [91]. Hardness and wear tests were performed to evaluate the effects of DCT and microstructural changes as a consequence of the treatment. DCT improves hardness and wear resistance of AZ91 by structure contraction, in which aluminum atoms in the β phase jump adjacent to the defects as new Mg17Al12 precipitates during ageing. As the expansion coefficient of α and β phase are different at a lower temperature, this difference is associated with different shrinkage values. As consequence of these different shrinkage values, dislocations and microvoids are generated on the boundary of the α and β phase that provide appropriate places for aluminum atoms to jump during DCT.

**Figure 11:**
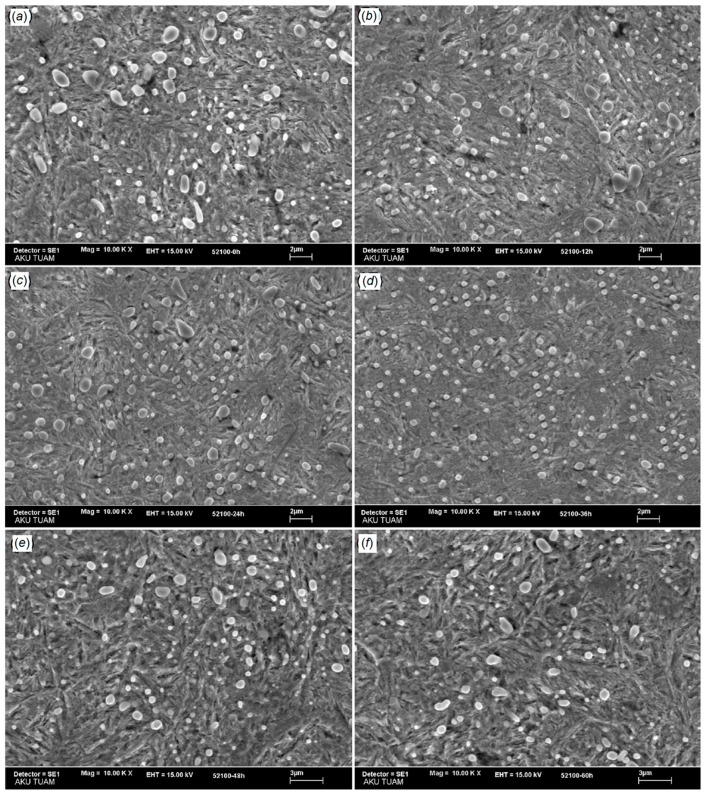
SEM microstructures of (**a**) CHT, (**b**) DCT-12 h, (**c**) DCT-24 h, (**d**) DCT-36 h, (**e**) DCT-48 h, and (**f**) DCT-60 h samples [92].

An investigation has been conducted to determine the impact of DCT on AISI 52100 bearing steel wear resistance [92]. Significant microstructural alteration and consequently higher wear resistance were obtained by DCT execution. Uniform carbide distribution and homogeneous particle size along the formation of new small size carbides were obtained after DCT, which resulted in higher wear resistance. Better hardness was achieved due to the redistribution of chromium carbides and the austenite to martensite transformation. It was highlighted that the soaking time is the most important process parameter to obtain a uniform carbide distribution, fine and homogeneous particle size, and better mechanical properties. Figure 11 provides evidence that until a certain duration of soaking time (12 to 36 h), positive effects on carbide particle size and distribution can be observed, but surprisingly, the opposite effects can be seen for a longer holding time.

The effects of different treatments on AISI D3, as-received, CH treated without tempering, and DC treated without tempering were evaluated [93]. Uniform distribution of primary and secondary chromium carbides with smaller sizes were promoted by CHT and DCT. The highest hardness was obtained by DCT followed by CHT and consequently better tribological performance was achieved by DCT.

Microstructure and wear properties of dies are essential parameters that affect the product quality and production costs. Therefore, the effects of treatment processes on the microstructure and wear resistance of AISI H13 hot-worked tool steel as the most common material in die production, have been examined [94]. Different treatment processes, conventional, DCT, and DCT plus tempering (DCTT), were considered and executed. It was noted that DCT encourages a homogeneous distribution of fine carbide particles in the martensite matrix and improves the wear resistance, but better wear resistance is obtained by DCT due to the formation of finer carbides with a more uniform distribution. In addition, tempering after DCT resulted in the precipitation of secondary carbides and improved their coherence with the matrix.

Li et al. performed experimental trials to study the effects of DCT on high-vanadium alloy steel microstructure and mechanical properties [95]. Enormous amounts of small secondary carbide precipitation and microcracks at the carbide and matrix interfaces were observed after execution of DCT. It was highlighted that the volume fraction of secondary carbides after DCT was found to be three to five times larger than those obtained by CT. This increment in the volume fraction of secondary carbides was found to be affected by the number of DCT cycles. In addition, DCT encourages the finer carbide particle size with a homogenous distribution. The DC treated sample formed smaller secondary spherical carbides. In contrast, as hardness decreases, toughness increases after DCT. A slight improvement of abrasive wear resistance was obtained after DCT execution, due to a larger amount of finer secondary carbide precipitation, which is affected by soaking time and the number of cycles.

An investigation was aimed at studying the effects of DCT on fracture toughness, wear resistance, and load-carrying capacity of cold-worked tool steel [96]. It was concluded that an improvement in the mechanical properties was as a consequence of finer, needle-like martensite formation and retained austenite transformation, combined with the plastic deformation of primary martensite. In addition, any alteration of fracture toughness to working hardness influences the wear resistance of cold-worked tool steel.

The effects of DCT and the correlation between microstructural alteration and wear behavior of WC–Fe–Ni cemented carbides has been examined and discussed [38]. Selective electrolytic corrosion technique was employed to study the phase composition and quantitative analysis of DCT. Wear resistance improvement was observed after execution of DCT due to the martensite phase transformation from γ-(Fe,Ni) to α-(Fe,Ni) and can be attributed to precipitation of W particles in the binder phase. It was highlighted that soaking time is the most important contributor to transformation of the binder phase from γ to α.

Li et al. applied DoE (Design of Experiment) orthogonal design to evaluate the effects of cryogenic-ageing circular treatment (CACT) on articles reinforced in-situ aluminum matrix composites [97]. Different combinations of cooling rate, soaking time and circular index as process variables were set. Al–Zn–Mg–Cu wrought aluminum reinforced by alumina particles, was selected as the host material to execute CACT. SEM analysis revealed that increasing the precipitation of Si phases improved the load carrying capacity of the metal matrix and consequently, the wear resistance. Hardness was reduced as a consequence of a reduction in the unstable but hard needle-like η’ (Zn_2_Mg) phase and an increment of the stable lamellar *h* (Zn_2_Mg) phase with low hardness.

In many mechanical applications, especially in automotive and aerospace engineering, as the components are reciprocating or rotating at relative speed to each other, wear resistance improvement is of paramount importance. The benefits of CT and its effects on the enhancement of wear resistance have been investigated and discussed in the analyzed literature as summarized by Table 2. However, the mechanisms responsible for the wear resistance improvement by DCT and SCT, in terms of microstructural alteration, are yet to be clarified. Therefore, the impacts of microstructural variation after CT on the wear resistance of steels can be summarized as below to correlate the wear behavior with microstructural alteration:

- Transformation of retained austenite to martensite, which is counted as a contributor to improve microhardness and enhancement of wear resistance to comply with it;
- Higher volume fraction of fine, and a homogeneous distribution of, carbide particles within the matrix resist against the dislocation movements and plastic deformation;
- Segregation of carbides and the formation of η-carbides improves the resistance against sliding wear as they reinforce the martensitic structure;
- Secondary carbide precipitation, especially during tempering;
- DCT is more efficient to enhance the wear resistance in comparison to SCT as it encourages a more uniform distribution of fine carbides and the elimination of retained austenite; soaking time and soaking temperature are the most effective process parameters.

In the case of cemented carbides, DCT enlarges grain size as a consequence of carbide grain contiguity and the dominant effect of the carbide phase, improving thermal conductivity and consequently, wear resistance. In addition, distribution of η-phase carbide within the structure empowers the bond between the carbide and binder, which brings higher wear resistance.

## 5. Effects of DCT on Microstructure and Mechanical Properties

In this section, the effect of CT on fatigue strength, tensile toughness, thermal conductivity, and durability, as well as residual stresses relief, will be addressed and discussed.

Bensely et al. conducted an experimental investigation to gain insight into fatigue and fracture behavior of carburized EN 353 steel subjected to CT [98]. Rotating bending fatigue tests were carried out to study the influences of SCT and DCT. It was highlighted that due to the presence of higher retained austenite and fine carbides, SCT treated specimens showed better fatigue life in contrast to DCT and conventionally treated specimens. A 71% improvement in fatigue life was observed via SCT and DCT.

Baldissera and Delprete studied the effect of DCT on tensile properties, fatigue life, and corrosion resistance of AISI 302 stainless steel in both hardened and solubilized states [99,100]. Although no significant effects on tensile strength were observed, changes in hardness and in elastic modulus were detected as far as significant increases in fatigue life for the solubilized material only. The fatigue fracture surface analysis pointed out that small secondary cracks for the treated material have been observed that could have acted as energy absorber with respect to the cyclic loading. The absence of significant effects from DCT applied to hardened AISI 302 was confirmed following an experimental investigation by Baldissera et al., where the same material was additionally subjected to CrN coating through Physical Vapour Deposition (PVD) [101].

The same authors carried out experimental investigations to assess the effects of DCT on 18NiCrMo5 carburized steel [102,103]. Two levels of temperature and soaking time were considered. In addition, the influence of the sequence among case hardening, tempering, and DCT was studied. Performing DCT before tempering produced a significant increase in hardness (+2.4 HRC), while pre-tempering DCT resulted in a remarkable enhancement of UTS (Ultimate tensile strength) (+11%). The most interesting result was observed in terms of scatter reduction of fatigue data, leading to a drastic increase in the fatigue limit at higher reliability levels (+25% considering a failure probability of 0.3%). In a subsequent study, the authors applied the Tanaka–Mura model for fatigue crack nucleation to the above experimental results through Maximum Likelihood Estimation (MLE), obtaining further insight on the potential causes of the observed improvements [104]. The conclusion was to address further investigations towards the effect of DCT on the specific fracture energy and, in agreement with most of the literature, on the dimensional and statistical characterization of subgrain precipitate fields.

Bouzada et al. evaluated microstructural alterations due to DCT of a heat-treated 7075 aluminum alloy [105]. Higher compressive residual stresses and slight changes in static mechanical properties (yield strength, tensile strength, and hardness) were observed after treatment. Accretion of submicrometric particles near and at the grain boundaries was detected. The combined effect of these microstructural changes improved the stress corrosion cracking and durability of 7075 aluminum.

The joint properties of 4 mm-thick 2219-T87 alloy produced by tungsten inert gas welding (TIG) and friction stir welding (FSW) at room temperature and DCT temperature (−196 °C) were examined. FSW presents better welding properties due to the preservation of the working structures and homogenous chemical compositions. A uniform and coherent microstructure obtained by DCT in the form of grain-boundary strengthening, substructure strengthening, and ageing precipitation strengthening is the main reason for the mechanical properties alteration [106].

The effect of microstructural alteration after DCT execution on tensile toughness in medium carbon-low alloy tool steel has been examined [107]. It was highlighted that the amount of secondary carbides is proportional to the soaking time. As soaking time increases, there was a constant increment of secondary carbides until a certain duration because after this point secondary carbide density is reduced. Tensile toughness was increased by simultaneously extending both the tempering duration and soaking time.

The combined effects of postweld heat treatment and low-temperature ageing treatment with and without DCT on friction-stir-welded joints of 2024-T351 aluminum alloys were examined [29]. It was observed that joint elongation increases with an improvement in tensile strength. Pre-DCT promotes the redissolution or dispersed precipitation of the unstable phases in the as-welded joints.

The effects of DCT on the fatigue behavior of 1.2542 tool steel have been examined [108]. It has been highlighted that nonsignificant changes in fatigue life were observed and both specimens were free of retained austenite. However, specimens that experienced DCT contained more secondary carbides with uniform distribution.

The effects of DCT on mechanical properties of induction hardened En 8 steel were measured, with the treatment carried out at −196 °C for 24 h [109]. A remarkable improvement of the ultimate tensile strength was observed as DCT increased the compressive residual stress of steel. SEM analysis provided evidence that the martensite structure was formed after DCT.

Araghchi et al. applied a combination of prior treatments, CT, and ageing (reheating by oil at 180 °C) on 2024 aluminum [110]. Application of this treatment cycle resulted in a reduction of residual stress by 250%. The concerned treatment encouraged the formation of large needle-like S´ precipitates with different orientation, which improved matrix hardness and reduced the residual stresses. It reported that S´ phase preferentially precipitated on dislocations.

An investigation was conducted to examine the effects of DCT, prior and after ageing, on microstructural evolution, microhardness, and tensile property variations of TB8 metastable β titanium alloy [111]. It was observed that conventional solution treatment followed by DCT resulted in a higher degree of super-cooling and internal stress through lattice shrinkage, which fosters the formation of needle-like α phase in the martensite matrix and furnishes a higher fraction of α phase during ageing treatment. The aforementioned microstructure alterations, as well as refinement and homogenization of lath-like α precipitates, improved the microhardness and tensile strength while reducing the elongation.

Subzero treatment was carried out on EN 1.4418 steel to evaluate the sensitivity of reverted austenite to temperature [112]. It was highlighted that impact toughness is a time dependent variable of temperature and it is directly affected by the volume fraction of austenite. Reverted austenite appears to be stable at room temperature and at elevated temperature (immersing in boiling N2), but in moderate temperature it can be partially transformed.

In general, according to the above literature survey, as summarized by Table 3, some important microstructural alterations due to CT and their effects on the mechanical properties are correlated with each other as follows:

- Residual stresses can be imposed by external or internal sources to the materials. Residual stresses due to external sources can be introduced and developed during manufacturing processes such as nonuniform plastic deformation under cold working, shot peening, hammering, grinding, or by welding due to thermal shocks. Some residual stresses are due to defects in the crystal structure of the materials, with the most common defects being in the form of vacancies, dislocations, and stacking faults. Residual stresses would cause creep failure, fatigue, and stress corrosion cracking in sensitive materials. The higher transformation of retained austenite to martensite at lower temperatures induces compressive residual stress, which is beneficial for wear resistance improvement and fatigue behavior. Precipitation of the carbides in DCT and SCT followed by tempering is the main contributor to residual stresses reduction;
- Fatigue life improvement due to CT is attributed to distribution of fine carbide particles and reduction of residual stresses. Moreover, the subgrain carbide distribution can play an important role by affecting the statistical dispersion of the fatigue behavior, with a significant impact at high reliability levels, which is the most interesting in many structural component applications;
- Fracture toughness after DCT reduced the transformation of retained austenite to martensite. DCT improves hardness and reduces fracture toughness. Therefore, it is essential to control the fracture toughness to avoid the risk of microcracking, especially in the case where mechanical components are sliding under the influence of a load in tribo-systems;
- Formation of martensite and the elimination of retained austenite, along with the precipitation of fine carbide, are the main contributors to the enhancement of ultimate tensile and yield strength.

## 6. Cryogenic Application in Manufacturing Engineering

Cryogenic treatment induces significant effects on engineering components and improves the mechanical properties through microstructural alteration. A major portion of manufacturing cost is contributed by tooling cost involving tool replacement and resharpening, material scrap, and production line interruption [113]. Improving cutting tool durability and workpiece machinability provides evidence to consider cryogenic treatment as a potential way to take a step toward sustainable manufacturing and clean production. In addition, in such applications where wear resistance is of paramount importance, such as tribo-systems and transportation applications, cryogenic treatment can improve the system reliability. In this context, to improve productivity, it is essential to develop tools with the capability to withstand higher cutting speeds and feed rates during machining. To achieve this goal, tool performance and machinability improvement of workpiece materials are of prime importance, especially in the case of hard to cut materials, as they are most in demand to operate under severe conditions [114,115]. Therefore, this section is devoted to addressing the application of cryogenic treatment in machining processes.

Yong et al. investigated the effects of cryogenic treatment on the cutting performance of tungsten carbide (G10E) during milling of ASSAB 760 medium carbon steel with and without the presence of coolant. The cryo-treated tool showed a better performance especially during wet milling, as DCT improves the thermal conductivity of the tool and promotes higher heat transfer at the tool/chip interface temperatures [116].

**Figure 12:**
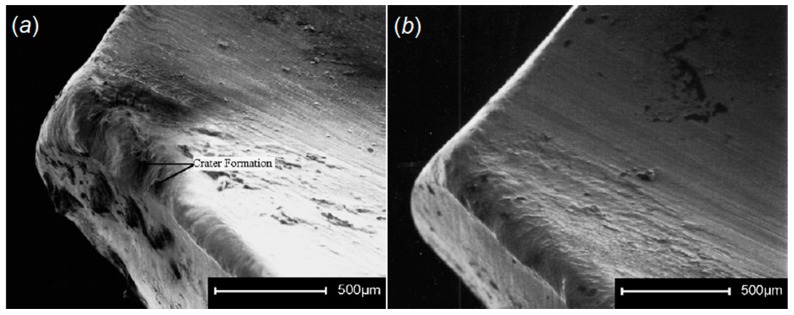
SEM images of drills after drilling of 30 holes: (**a**) nontreated drill and (**b**) cryo-treated with tempering drill [117].

The effects of CT on wear resistance and tool life of M2 HSS drills during high speed dry drilling of normalized CK40 carbon were measured. The performance of three different tools, untreated, cryo-treated, and cryo-treated followed by tempering were examined. The findings revealed that cryo-treated drills showed better wear resistance to diffusion wear due to the formation of a higher volume fraction of fine and homogeneous carbide particles in the matrix as demonstrated by Figure 12 [117].

**Figure 13:**
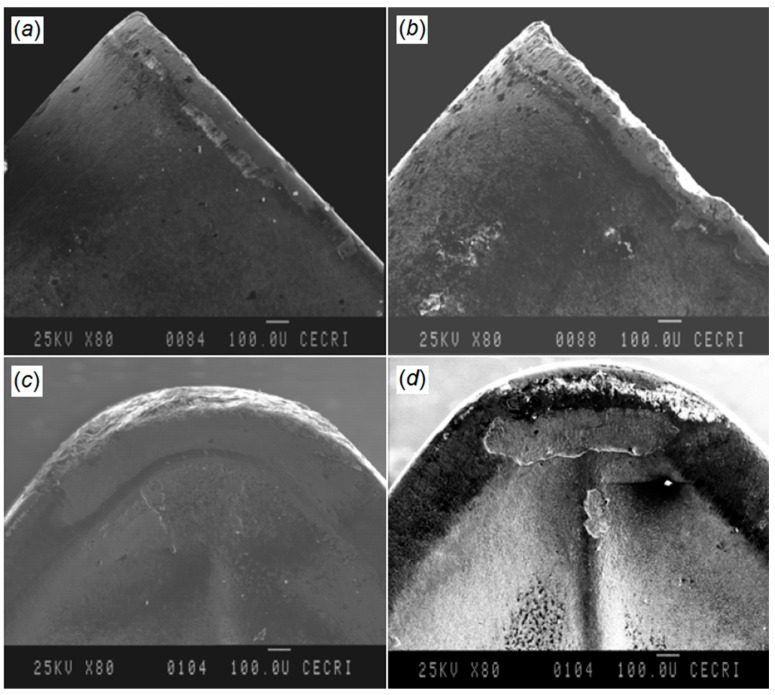
Flank and crater wear of cryo-treated (**a**,**b**) and non-treated (**b**,**d**) inserts [118].

Tool wear and power consumption were considered as indicators to evaluate coated carbide inserts subjected to CT during turning of AISI/SAE 80-55-06 SG iron. Cryo-treated inserts provided better performance due to a reduction of the η-phase (carbide) effect on the hardness and wear resistance by CT, as it promotes a uniform distribution of refined carbide particles and their denser and more coherent bonding with the matrix. Figure 13 shows flank and crater wear of cryo-treated (a and c) and untreated (b and d) cutting tools at a cutting speed of 250 m/min, feed rate of 0.1 mm/rev, and depth of cut 0.8 mm [118].

**Figure 14:**
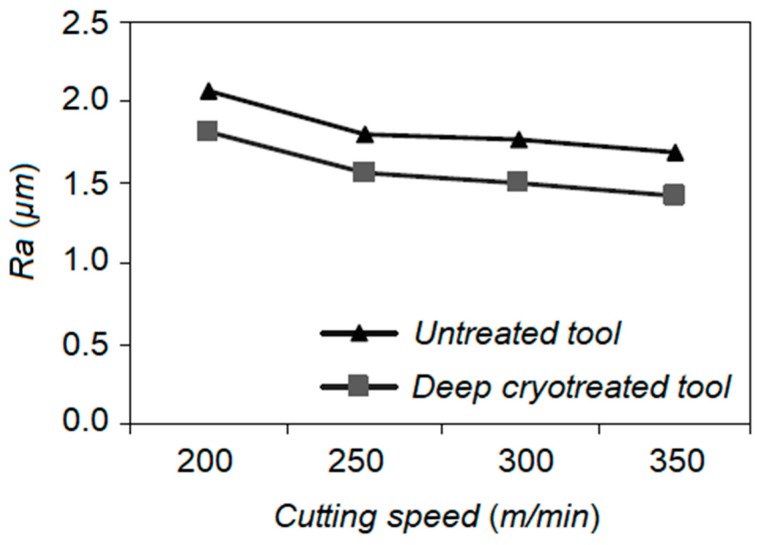
Surface roughness at different cutting speeds of P-30 insert [119].

SreeramaReddy et al. examined the effects of DCT on tungsten carbide cutting tool inserts performance during machining of C45 steel [119]. Cutting tool flank wear, cutting force, and surface roughness were set as indicators to evaluate the machinability of C45 and deep cryogenic treated tungsten carbide inserts. The cutting tool durability after DCT was improved as the hardness increased in comparison to untreated inserts. A better surface was obtained using a DCT treated tool as the wear resistance of the insert was increased and a negligible alteration in the cutting edge geometry was observed (Figure 14). An increment in the carbide grain size after DCT increased the thermal conductivity of the cemented carbide as a result of an increase in carbide grain contiguity and the dominant effect of the carbide phase. This impact reduces the tool tip temperature and improves its resistance against the erosion.

The machinability of coated tungsten carbide tool inserts of ISO P-40 exposed to DCT has been examined in terms of wear resistance, cutting force, and surface roughness [120]. Turning was carried out on AISI 1040 steel using untreated and cryo-treated (−176 °C) cutting tools. DCT improves the thermal conductivity of the insert and the cutting zone temperature is dissipated by the chips and consequently encourages lesser tool wear. Higher thermal conductivity of the cutting tool plays an important role at higher cutting speeds. This results in massive seizure through the chips and enables the discontinuous chips, which improves tool life by reducing the adhesive wear rate of cutting edges leading to better surface finish [121].

Sundaram et al. applied DCT to improve the mechanical and electrical properties of copper tool electrodes, while electrical discharge machining (EDM) of Be–Cu. A higher material removal rate (MRR) was obtained by the cryo-treated electrodes due to an improvement of electrical conductivity, but the impact on the electrode wear rate (EWR) was marginal [122].

Cryogenically treated copper electrodes were employed to study the effect of DCT during the electrical discharge drilling (EDD) of Ti-6246. Hole quality in terms of surface roughness and overcut was examined to compare cryo-treated copper electrode performance with the as-received one. MRR and EWR produced surface finish, and the hole quality using cryo-treated electrodes was significantly improved [123].

Gill et al. conducted an experimental investigation to examine the effects of SCT and DCT on tungsten carbide inserts (P25) during dry turning of hot-rolled annealed steel stock (C-65). Maximum flank wear (ISO 3685-1993) and surface roughness were considered as criteria to determine the turning performance. Longer durability was recorded for the DCT insert followed by SCT tools as compared to untreated ones. Cryogenic treatment causes higher precipitation of η-phase carbides within the matrix and enhances the carbide tool life [124].

The performance of cubic boron nitride (CBN) inserts in terms of flank wear, surface finish, white layer formation, and microhardness were compared against cryo-treated coated/uncoated carbide cutting tools during dry turning of hardened AISI H11 steel. The surface roughness produced by cryo-treated inserts were comparable with that generated by CBN tools. In addition, cryogenically treated inserts provide longer tool life with a 16–23% improvement [125].

Longer tool life and 35% reduction in surface roughness (Ra) were reported using cryo-treated AISI M2 tools during drilling of mild steel C45 as results of wear resistance improvement, and a reduction in the anisotropy of the tool. Cryogenic temperature and soaking time were the most influential parameters, respectively [126].

The performance of cryogenically treated M35 HSS under dry drilling of stainless steels has been investigated. Better wear resistance and longer tool life were obtained due to the transformation of retained austenite to martensite, higher volume fraction, and more homogenous distribution of carbide particles [127].

Experimental trials were conducted to evaluate the performance of cryo-treated copper electrodes and machining EDM using a dielectric mixed with graphite powder additive while machining Inconel 718. MRR and EWR were improved using the cryo-treated copper electrode, due to the formation of hard carbide compounds on the electrode caused by the migration of carbon from the decomposed dielectric fluid and refinement of the grains, which improved the electrical and thermal conductivity [128]. The addition of metal powder in the dielectric improved the machining efficiency by making discharge breakdown easier and enlarging the discharge gap [129].

The effects of CT on surface residual stresses of ground carbide surfaces after grinding using diamond abrasive wheel were examined. Thermal residual stresses were induced due to thermal mismatch between the WC and the Co phase. A 20% reduction in residual stresses after CT execution was observed due to the cracking and plastic deformation in the WC grains [130].

A significant improvement of the cutting performance of cryo-treated M35 HSS during drilling of AISI 316 austenitic stainless steel was observed in comparison to untreated drills. Tool life, surface roughness, and hole quality were selected as criteria to assess the tool life. The improvements were attributed to the transformation of retained austenite into martensite and a more homogeneous distribution of carbides due to secondary carbide precipitation [131].

Kapoor et al. examined the performance of deep cryo-treated brass electrodes during EDM of En-31. Significant improvement of the electrical conductivity and MRR after DCT was observed due to refinement of grains and a reduction in microcavities [132]. It is worth mentioning that the electrical conductivity is proportional to the thermal conductivity, therefore, the higher the electrical conductivity, the higher the MRR [133,134].

Çiçek et al. examined the effect of CHT, CT, and CTT on the machinability of AISI H13, subjected to turning using CC670 and CC650 ceramic inserts, in terms of tool wear, surface roughness, and cutting force. A better surface quality for the CCT workpieces was obtained, followed by the CT and CHT specimens. The cryo-tempering process reduces the workpiece hardness and encourages a lower cutting force, tool wear, and surface roughness, due to the formation of higher volume fraction of fine carbides [135].

The effects of DCT on the machining efficiency of microelectric discharge machining (MEDM) of AISI 304, using copper, brass, and tungsten electrodes were evaluated. Lower EWR was obtained using cryo-treated tungsten followed by the brass and copper electrodes with a reduction of 58%, 51%, and 35%, respectively. A higher volume fraction of refined particles was observed within the brass matrix after execution of DCT as compared to copper and tungsten. DCT increases average crystal size of brass, copper, and tungsten by the value of 29%, 12%, and 4%, respectively, which results in the enhancement of hardness and wear resistance [136].

The effects of different soaking times on durability (wear resistance) of deep cryogenic treated AISI 316 austenitic stainless steel has been studied. Wear tests were conducted at different cutting speeds with a constant feed rate and depth of cut under dry turning. An improvement of the wear resistance of the cutting tool was observed. However, flank wear and crater wear were presented as a result of new η-carbide particles in the microstructure formation and a uniform and homogeneous distribution of small-sized carbide particles [137].

Mavi and Korkut examined the effect of CT on the performance of cemented carbide during turning of Ti-6Al-4V. An increment of 22% of cryo-treated inserts was reported as a consequence of fine and uniform distribution of carbide particles within the matrix. Higher thermal conductivity of the cryo-treated inserts reduces cutting zone temperature and facilitates chip removal and consequently, a lower cutting force and better surface roughness were obtained [138].

Three different grades of titanium alloy, Ti (grade two), Ti-6Al-4V (grade five), and Ti-5Al-2.5Sn (grade six) subjected to SCT and DCT, were machined by EDM. Copper, copper–chromium, and copper–tungsten electrodes were used to get a comprehensive insight into the cryogenic treatments impact. Marginal improvement of MRR was observed [139].

The effects of DCT on the machinability and wear behavior of TiAlN coated tools under dry turning of AISI 5140 have been studied [140]. These influences were examined by means of cutting force, cutting zone temperature, surface texture, and the tool life. Strengthening of the TiAlN coating on the tungsten carbide, improvement of substrate interfacial adhesion bonding, homogeneous carbide distribution, higher hardness, and better thermal conductivity were obtained after DCT. Therefore, DCT coated inserts show better machinability in comparison to uncoated, conventionally treated cutting tools.

Wear resistance and tool life of H13A tungsten carbide inserts subjected to DCT during turning of AISI 1045 steel has been investigated. Deep cryo-treated H13A provided higher hardness, better abrasive wear resistance, and lower toughness. DCT promotes a higher volume fraction of the cobalt phase without swelling of the tungsten carbide grains and strengthens the bonding between the tungsten carbide grains and the cobalt binder, which improves inserts corrosion resistance. It was highlighted that built up edge (BUE) was the predominant mechanism of rake surface adhesion wear of the cryo-treated inserts [141].

The effects of post treatment processes, cryogenic treatment, controlled heating, and oil quenching on cutting performance of cemented tungsten carbide (K20) under turning of Inconel 718 superalloy have been examined. Higher microhardness was obtained by oil quenched treatment following CT, as the quenching increases the stresses imposed on the carbide phase (compressive stresses) and the binder (tensile stresses) with decreasing cobalt content, which reduces ductility. In contrast, CT tends to relieve the stresses induced during the sintering process that is used to produce carbide tools, which improves the hardness in comparison to untreated inserts. In addition, CT encourages more densification of the cobalt binder which is strongly bonded by tungsten carbides and improves wear resistance and reduces cutting force [142].

The influences of different treatments on grindability of 9Mn2V using a grinding wheel (3SG80KV) have been studied. Four treatment process were conducted: 1) quenching followed by oil cooling; 2) quenching followed by oil cooling and tempering; 3) quenching followed by oil cooling, CT, and tempering; and 4) quenching followed by oil cooling, CT, and two times tempering. Cryogenic treatment promotes the uniform distribution of martensite, a higher volume fraction of refined carbides, and the transformed austenite to martensite. Carbide segregation occurred in all specimens but in the last two treatments comprising CT, carbide segregation was improved after CT and tempering. Machinability of two last samples was significantly improved due to higher volume fraction and uniform distribution of secondary carbides. In addition, impact toughness improvement and residual stresses relief on the workpieces surface due to CT and tempering, especially for the cryo-treated sample followed by double tempering, were detected [143].

The cutting capabilities of M2 HSS drill subjected to CT, CTT, and TiAlN/TiN-coated were evaluated while drilling Ti-6Al-4V under dry and wet conditions. Cryo-treated drills show higher wear resistance as compared to untreated and TiAlN/TiN-coated. After CT execution, the tool microstructure received finer carbide particles with a homogenous distribution, as well as a higher volume fraction of martensite, which encourages longer tool life in comparison to untreated drills. Cryogenic treatment is more cost effective than coating and brings fairly remarkable improvements [144].

Özbek et al. evaluated uncoated and deep cryo-treated tungsten carbide insert’s performance while turning an austenitic stainless steel (AISI 316). Flank, notch, and crater wear were selected as indicators to examine the tool life. A higher volume fraction of fine η-carbides after DCT execution, improved the hardness and wear resistance. DCT encourages larger grain size and better thermal conductivity which improved the flank wear by 34% and the crater wear by 53% [145].

Xu et al. examined the effects of DCT on the residual stresses and mechanical properties of electron beam (EB)-welded Ti-6Al-4V (TC4) joints [146]. Soaking time influence was evaluated to determine an optimum time. It was highlighted that a reduction in longitudinal and transversal residual stress (with respect to welding direction) after DCT was different. It was observed that the hardness in the welded area was higher than that in the base metal, and the average values of hardness increased with DCT due to the alteration of quantity, size, and morphology of the α and β phases.

Khanna and Singh studied the impact of DCT on machining efficiency using a high chromium cold alloy tool (D3) during WEDM (wire-cut electrical discharge machining). MRR was reduced by 5.6% after DCT due to an improvement of thermal conductivity as result of the transformation of retained austenite to martensite and the refinement of carbide particles, which promotes the heat flow laterally across the line of the cut [147].

The machinability of the AISI D2 tool steel subjected to DCT during EDM using copper electrodes has been investigated. Higher MRR (~18% enhancement), lower EWR, and surface roughness (26% and 11% improvement, respectively) were obtained after DCT as compared to untreated workpieces [148].

The performance of cutting inserts PVD AlTiN-coated (KC5525) and uncoated (K313) subjected to the DCT during dry turning of Nimonic 90 alloy were investigated. DCT promotes the formation of η-phase carbide particles and grain refinement which results in higher hardness and wear resistance. In addition, DCT strengthens the coating and reduces the failure probability in comparison to coating durability and damage of untreated inserts [149].

Khan et al. used cryo-treated K313 inserts during dry turning of CP-Ti grade two to evaluate the effects of DCT on tool performance. DCT increased the microhardness, wear resistance, and improved the chip formation phenomena such as chip compression ratio and friction coefficient. An increment of η-phase carbides is to be recognized as the main contributor to these improvements [150].

Naveena et al. carried out experimental trials to evaluate cryogenically treated tungsten carbide under drilling of AISI 304. Surface roughness using cryo-treated tools was reduced by 40% due to the enhancement of wear resistance and cutting edge stability. DCT encouraged a 19% reduction in average grain size of the α-phase and consequently, increased the hardness. Furthermore, DCT reduced the volume fraction of β-phase Co which implied combination of cobalt with WC particles to form η-phase carbides [151].

The research works devoted to examining the effects of CT on machinability of different materials subjected to nonconventional and conventional manufacturing process have been tabulated by Table 4 and Table 5, respectively.

## 7. Conclusions

A literature survey has been conducted to focus on cryogenic treatments effects on microstructural alteration and its correlation with material mechanical properties and manufacturability. Comparative investigations have been made to evaluate the effects of CHT, SCT, and DCT on the microstructure and mechanical property variations. The effects of important parameters and their interactions such as austenitizing temperatures, ramp down, soaking time, and temperature, as well as ramp up, are discussed. The review of the literature implies that the evaluation of microstructural variations due to cryo-treatment and their interaction with mechanical properties is a complex phenomenon. Therefore, these complexities of interactions of microstructural alteration and mechanical properties depend on the adopted methods such as prior and supplementary treatments (austenitizing, quenching, and tempering), influential parameters, and demanded accuracy.

A review of the literature and the synthesis of technical contributions indicates various ways to execute cryogenic treatments. The important conclusions of this literature survey and the future directions are suggested as follows:

- In almost all steels, elimination of retained austenite, the increment of carbide volume fraction with homogenous distribution, and uniform size, as well as the formation of fine secondary carbide precipitation, are the greatest contributor to hardness improvement after DCT. Microstructure during SCT experiences these changes with a lower magnitude in comparison to DCT, but more efficiently than CHT;
- Uniform carbide distribution and homogeneous particle size, along with the formation of new small size carbides, have been obtained after DCT which result in higher wear resistance. Meanwhile, the formation of η-carbide and a homogeneous distribution of the produced carbides are the most important players in wear resistance improvement, rather than only the elimination of retained austenite. In the case of CT of steels, to obtain better properties and to improve treatment efficiency, it is recommended that austenizing, quenching, DCT, and tempering are carried out one after another in a cycle;
- Homogenous distribution of secondary carbides and the transformation of retained austenite to martensite after SCT and DCT improve fatigue strength and tensile toughness;
- Applying appropriate cooling temperature and soaking time causes substantial transformation of the soft austenite into hard martensite and in addition, metallurgical alteration of the martensite can be obtained;
- It is relevant to note that martensitic transformation never goes to completion after DCT, and the retained austenite always exists in the structure of high-carbon martensite. However, further tempering is suggested to boost the elimination of austenite and the formation of secondary carbides within the martensite;
- DCT encourages the increment of carbide grain size and improves the thermal conductivity of cemented carbide cutting tools. Therefore, as consequence of the increment in carbide grain contiguity, and the dominant effect of the carbide phase, higher wear resistance, and better surface roughness can be obtained;
- It is recommended to standardize the CT cycles including ramp down, soaking time and temperature as well as ramp up and supplementary processes, to optimize the properties of each specific material. Process qualification and standards would be drafted and validated for more promising alloys;
- The effects of CT on different cutting tools needs to be clarified and standardized to improve the performance of inserts in terms of wear, hardness, dimensional stability, and thermal conductivity.
- Special efforts are required to characterize the correlation between microstructural alterations and wear mechanisms of cutting tools from different materials.

## Figures and Tables

**Table 1:** Summary of literature data devoted to studying the effects of cryo-treatment on microstructure and hardness.

First Author, [#]	Cryogenic Treatment	Rival Treatment	Material	Microstructure Alteration	Outcome
Amini [34]	DCT	N.A.	1.2080 tool steel	Elimination of retained austenite, increment of carbide particles density	Hardness improvement was obtained.
Zhirafar [40]	DCT	N.A.	AISI 4340	Austenite transformation into martensite	Hardness and fatigue limit were improved.
Vimal [41]	DCT followed with tempering	N.A.	En 31 bearing steel	Austenite to martensite transformation coupled with higher volume fraction of fine carbides	Hardness improvement
Harish [42]	SCT and DCT	N.A.	En 31 bearing steel	Distribution of medium size spheroidized carbide particles and exitance of retained austenite even after DCT and SCT	Higher hardness obtained after DCT followed by SCT
Li [43]	DCT	Quenching and tempering	Die steel (Cr8Mo2SiV)	Martensite and austenite lattice contraction and homogeneous carbide distribution	Higher hardness obtained by DCT
Jeleńkowski [44]	Quenching+ DCT+ tempering	N.A.	HS6-5-2	Obtained martensite with lamellar-lenticular structure, and internally twinned, with very high density of dislocations as well as homogeneous distribution of spherical carbides	Hardness improvement
Senthilkumar [45]	SCT and DCT	N.A.	4140 steel	Reduction in lattice defects after DCT and residual stress relief in comparison to quenching+ SCT	Hardness improvement and residual stress releasement
Mehtedi [46]	DCT	N.A.	X30 CrMoN 15 1 steel	transformation of the retained austenite to martensite and homogeneous decoration of martensitic matrix by refined carbides particles	Higher hardness was recorded.
Candane [48]	SCT and DCT	CHT	AISI M35 HSS	Higher reduction in volume fraction of retained austenite was obtained by DCT followed by SCT.	Better hardness obtained by DCT followed by SCT
Akhbarizadeh [49]	DCT+ tempering	N.A.	1.2080 tool steel	Carbon atoms segregation and carbide nucleation	Hardness improvement
SreeramaReddy [50]	DCT	N.A.	WC–12Co cemented carbide	Transformation of ɛ-Co	Improvement of hardness and bending strength of cemented
Idayan [52]	SCT and DC	CHT	AISI 440C bearing steel	Higher rate of retained austenite transformation into martensite was achieved by DCT followed by SCT	Higher hardness was obtained by DCT.
Xie [53]	DCT	N.A.	WC−11Co cemented carbides	DCT refines WC grains into triangular prism with round edges without size alteration through the spheroidization process	DCT improved hardness and bending strength of the alloys.
Yuan [54]	DCT	N.A.	Pure zirconium	DCT reoriented grain is much closer to (0 0 0 1) basal plane	Increment in material resistance against plastic deformation and improving the hardness
Pérez [55]	DCT	N.A.	H3 tool steel	Reduction in retained austenite content	H3 steel hardness decreased, as carbide precipitation and carbon content of the martensite reduced.
Mohan [56]	DCT	N.A.	Al7075-T6	Precipitation, better distribution of second-phase particles, and higher dislocation density	Hardness and fatigue limit improved.
Nazarian [57]	SCT and DCT	N.A.	Al2024	Grain size and formed nanoparticles in microstructure were refined.	Hardness and fatigue limit were reduced as formation of microcracks.
Taşkesen [58]	DCT	N.A.	Al7075	Smaller atomic distance, refined particle shape and uniform microstructure	DCT encouraged higher friction against dislocation movement and higher hardness.

**Table 2:** Summary of the literature data devoted to studying the effects of cryo-treatment on microstructure and wear resistance.

First Author, [#]	Cryogenic Treatment	Rival Treatment	Material	Microstructure Alteration	Outcome
Thakur [59]	DCT	N.A.	Tungsten carbide–cobalt	Densification of the cobalt metal binder	Wear resistance has been improved.
Akhbarizadeh [60,62,72]	SCT and DCT	N.A.	D6 tool steel and 1.2080 tool steel	Higher volume of homogenized carbide distribution and elimination of the retained austenite	DCT homogenizes microstructure which encourages higher wear resistance
Dhokey [61]	DCT	Quenching and tempering	D3 tool steel	Higher volume fraction of fine carbides and their nucleation during ramp up	Wear resistance improvement.
Das [63,68,85,87]	SCT and DCT	DHT	AISI D2	Reduction in retained austenite and higher volume fraction of secondary carbides	Wear behavior is proportional to secondary carbides morphology. DCT is most effective treatment.
Straffelini [64]	DCT	AlCrN PVD coating and	Stamping tools	Precipitation of ultrafine carbide particles	Wear behavior was improved.
Podgornik [65,88,97]	DCT	N.A.	Powder–metallurgic (PM) high-speed steel and Cold-work tool steel	Homogenous microstructure, finer needles like martensite formation and retained austenite elimination	Abrasive wear resistance has been enhanced.
Wang [66]	DCT	N.A.	High chromium cast iron	Transformation of abundant retained austenite into martensite and finer secondary carbide precipitation	Wear resistance improvement was recorded.
Oppenkowski [67]	DCT	N.A.	AISI D2	Transformation of retained austenite into martensite	Transformation of retained austenite to martensite is affected by soaking time and temperature.
Cajner [69]	DCT	N.A.	PM S390 MC high speed steels	Fine η-carbides formed within the matrix.	Wear behavior was improved.
Asl [70]	DCT	N.A.	Magnesium alloy AZ91	Alteration of β precipitates distribution, coexistence of dissolved tiny laminar β particles and coarse divorced eutectic β phase	Wear resistance was enhanced.
Sogalad [71]	DCT	N.A.	En8 steel	Transformation of retained austenite to martensite and distribution of fine η-carbides	Wear resistance and load carrying capacity were improved.
Siva [73]	DCT	CHT	100Cr6 bearing steel	Martensite destabilization by triggering carbon clusterization and carbide precipitation	Wear resistance and hardness were improved.
Xu [74]	DCT	N.A.	AISI H13	Homogeneous martensite	Wear resistance was enhanced.
Arockia Jaswin [75]	DCT	N.A.	E52 valve steel	Transformation of retained austenite to martensite and increase in the amount of fine secondary carbide precipitation	Wear behavior was ameliorated.
Li [76]	DCT + tempering	N.A.	Tool steel	Precipitation of carbide particle and carbon segregation nearby dislocation and carbon cluster formation	Improvement of wear resistance was highlighted.
Koneshlou [77]	DCT	N.A.	H13 tool steel	Uniform distribution of martensite laths and transformation of retained austenite to martensite	Wear properties has been improved.
Senthilkumar [78]	SCT and DCT	CHT	En19 steel	Precipitation of fine carbides and transformation of retained austenite into martensite	Higher wear resistance was obtained after DCT followed by SCT.
Jaswin [79]	SCT and DCT	CHT	X53Cr22Mn9Ni4N valve steels	Elimination of the retained austenite and formation of fine carbides	Wear resistance was improved.
Li [80]	DCT	N.A.	Cold work die steel	Carbon atoms segregation and generation of strong interaction with dislocations	Wear resistance improvement
Amini [81,86]	DCT	N.A.	80CrMo12 5 tool steel, D2 tool steel	Elimination of retained austenite, higher amount, and fine distribution of carbide	Wear resistance and ultimate tensile strength were improved.
Gill [82]	SCT and DCT	N.A.	AISI M2 HSS	Transformation of austenite into martensite and higher precipitation of small carbides	Wear behavior was ameliorated.
Sri Siva [83]	DCT	N.A.	100Cr6 bearing steel	Precipitation of the fine carbides and transformation of the retained austenite to martensite	Wear resistance, hardness, and dimensional stability were improved.
Gavriljuk [84]	DCT	N.A.	Steel X153CrMoV12	Refining of primary and secondary carbides	Abrasive wear resistance and hardness were enhanced.
Amini [91]	DCT	N.A.	AZ91 magnesium alloy	Aluminum atoms in the β phase jump adjacent to the defects	Wear and hardness were improved.
Gunes [92]	DCT	N.A.	AISI 52100	Uniform carbide distribution, refinement of particle size and redistribution of chromium carbides	Higher wear resistance was obtained.
Khun [93]	DCT	CHT	AISI D3	Uniform distribution of primary and secondary chromium carbides	Higher wear behavior and hardness were recorded.
Çiçek [94]	DCT and DCTT	N.A.	AISI H13	Finer carbides size and distribution	Wear resistance was improved. Tempering after DCT resulted in precipitation of secondary carbides.
Li [95]	DCT	N.A.	High-vanadium alloy steel	Finer carbide particles size with homogenous distribution	Higher abrasive wear resistance was obtained.
Li [97]	DCT	N.A.	Al–Zn–Mg–Cu wrought aluminum	Precipitation of Si phases and reduction in unstable but hard needle-like η’ (Zn2Mg) phase	Wear performance and hardness were improved.

**Table 3:** Summary of literature data devoted to study the effects of cryo-treatment on microstructure and mechanical properties.

First Author, [#]	Cryogenic Treatment	Rival Treatment	Material	Microstructure Alteration	Outcome
Bensely [98]	SCT and DCT	N.A.	EN 353	Higher volume fraction of retained austenite and fine carbides	SCT treated specimens show better fatigue life in contrast to DCT and CHT.
Baldissera [99,100,101]	DCT	CrN coating through PVD	AISI 302	Formation of microsecondary cracks on surface	Tensile strength and fatigue life significantly improved.
Baldissera [102,103,104]	DCT	N.A.	18NiCrMo5 carburized steel	N.A.	Hardness (+2.4 HRC) and UTS (+11%) were improved.
Bouzada [105]	DCT	N.A.	7075 aluminum alloy	Accretion of submicrometric particles near and at the grain boundaries	Yield strength, tensile strength and hardness were ameliorated
Lei [106]	DCT	N.A.	2219-T87	Uniform grain-boundary strengthening, substructure strengthening, and aging precipitation strengthening	Better weldability obtained.
Vahdat [107]	DCT	N.A.	Medium carbon-low alloy tool steel	Formation of secondary carbides	Tensile toughness increased.
Niaki [108]	DCT	N.A.	1.2542 tool steel	Elimination of retained austenite and uniform distribution of secondary carbides	Nonsignificant changes in fatigue life were recorded.
Senthilkumar [109]	DCT	N.A.	En 8 steel	Uniform martensite structure	Significant improvement of ultimate tensile strength was traced.
Araghchi [110]	DCT	N.A.	2024 aluminum	Formation of large needle-like S´ precipitates with different orientation	Reduction of residual stress by 250% and hardness increase have been reported.
Gu [111]	DCT	N.A.	TB8 metastable β titanium alloy	High volume fraction of needle-like α phase in martensite matrix	Microhardness and tensile strength were improved.
Nießen [112]	DCT	N.A.	EN 1.4418 steel	Stabilization of reverted austenite	Impact toughness is a time dependent variable on temperature, and it is directly affected by volume fraction of austenite.

**Table 4:** Literature data related applying cryogenic treatment in nonconventional manufacturing process.

First Author, [#]	Process	Tool Material	Workpiece Material	Key Findings
Sundaram [122]	EDM	Copper	Be–Cu	Higher MRR due to electrical conductivity improvement, and marginal effect on electrode life
Gill [123]	EDD	Copper	Ti-6246	DCT improves MRR, electrode life, and surface finish.
Kumar [128]	EDM	Copper	Inconel 718	MRR and EWR have been improved due to formation of hard carbide compounds on the electrode.
Kapoor [132]	EDM	Brass	En-31	Improvement of electrical conductivity and MRR after DCT has been achieved due to refinement of grains and reduction in microcavities.
Jafferson [136]	MEDM	Copper	AISI 304	DCT encourages average crystal size of brass, copper, and tungsten by the value of 29%, 12%, and 4%, respectively, which result in enhancement of hardness and wear resistance.
Kumar [139]	EDM	Copper and Copper-Tungsten	Ti, Ti-6Al-4V, and Ti-5Al-2.5Sn	Marginal improvement of MRR was observed.
Xu [146]	EB welding	N.A.	Ti-6Al-4V	Due to alteration of quantity, size, and morphology of the α and β phases after DCT, hardness in welded area was higher than that in the base metal.
Khanna [147]	WEDM	N.A.	AISI D3	Transformation of retained austenite to martensite and refinement of carbide particles after DCT execution, thermal conductivity has been improved.
Goyal [148]	EDM	Copper	AISI D2	Higher MRR (~18% enhancement), lower EWR, and surface roughness (26% and 11% improvement, respectively) were obtained after DCT.

**Table 5:** Literature data related applying cryogenic treatment in conventional manufacturing process.

First Author, [#]	Process	Tool Material	Workpiece Material	Key Findings
Yong [116]	Milling	Tungsten carbide G10E	ASSAB 760	DCT improves tool life as consequence of higher heat transfer.
Firouzdor [117]	Drilling	M2 HSS	CK40	Better wear resistance against diffusion wear.
Vadivel [118]	Turning	Coated carbide inserts	AISI/SAE 80-55-06 SG	Higher hardness and better wear performance due to uniform distribution and higher volume fraction of refine carbides particles.
SreeramaReddy [119]	Turning	Tungsten carbide	C45 steel	Increment of carbide grain size after DCT increased the thermal conductivity and reduced cutting tool tip temperature.
Reddy [120]	Turning	ISO P-40	AISI 1040	Lower tool wear due to thermal conductivity improvement after DCT has been observed.
Gill [124]	Turning	P25	C-65	Longer durability was recorded for DCT insert followed by SCT due to higher precipitation of η-phase carbides.
Dogra [125]	Turning	Cubic boron nitride (CBN)	AISI H11	16%–23% tool life improvement has been reported.
Shirbhate [126]	Drilling	AISI M2	C45	Longer tool life and 35% reduction in surface roughness (Ra) were reported after DCT execution.
Çiçek [127]	Drilling	M35 HSS	Stainless steels	Longer tool life was obtained due to transformation of retained austenite to martensite and homogenous distribution of carbides particles.
Yuan [130]	Grinding	Diamond abrasive wheel	Ultra-fine grade cemented carbide	20% reduction in residual stresses after CT execution were observed due to the cracking and plastic deformation in the WC grains.
Çiçek [131]	Drilling	M35 HSS	AISI 316	Transformation of retained austenite into martensite and more homogeneous distribution of carbides provided better tool performance.
Çiçek [135]	Turning	Ceramic Inserts	AISI H13	DCT reduces tool wear and surface roughness as result of higher volume fraction of fine carbides formation.
Özbek [137]	Turning	Cemented carbide	AISI 316	Tool life was improved due to homogeneous distribution of small-sized carbide particles.
Mavi [138]	Turning	Cemented carbide	Ti-6Al-4 V	Tool life has been improved by 22% due to higher thermal conductivity improvement.
He [140]	Turning	Tungsten carbide	AISI 5140	DCT coated inserts were shown better machinability in terms of cutting force, cutting zone temperature, surface texture, and tool life.
Thornton [141]	Turning	H13A	AISI 1045	Better corrosion resistance obtained due to strengthen of carbide grains and the cobalt binder.
Thakur [142]	Turning	K 20	Inconel 718	CT encourages more densification of the cobalt binder which is strongly bonded by tungsten carbides and improves tool wear resistance.
Dong [143]	Grinding	Grinding wheel (3SG80KV)	9Mn2 V	Improvement and releasement of residual stresses on the workpieces surface has been improved.
Kivak [144]	Drilling	M2 HSS	Ti-6Al-4 V	It was concluded that CT is more cost effective than coating which brings remarkable improvements.
Özbek [145]	Turning	Tungsten carbide	AISI 316	Higher volume fraction of fine η-carbides after DCT execution improves the hardness and wear resistance.
Chetan [149]	Turning	KC5525 and K313	Nimonic 90	DCT strengthens the coating and reduces failure probability in comparison to coating durability and damage on untreated inserts
Khan [150]	Turning	K313	CP-Ti grade 2	DCT increases microhardness, wear resistance and improves chip formation phenomenon.
Naveena [151]	Drilling	Tungsten Carbide	AISI 304	DCT encourages 19% reduction in average grain size of α-phase and consequently increases the hardness and improves wear resistance.

## Nomenclature

Conventional heat treatment	CHT
Cryogenic treatment	CT
Cryogenic treatment followed by tempering	CTT
Cryogenic-ageing circular treatment	CACT
Deep cryogenic treatment	DCT
Deep cryogenic treatment pluse tempering	DCTT
Differential scanning calorimetry	DSC
Electron back scattered diffraction	EBSD
Electrical discharge machining	EDM
Electrode wear rate	EWR
Electric discharge drilling	EDD
Friction Stir Welding	FSW
Microelectric discharge machining	MEDM
Material removal rate	MRR
Maximum Likelihood Estimation	MLE
Optical microscopy	OM
Subzero treatment	SZT
Shallow cryogenic treatment	SCT
Scanning electron microscopy	SEM
Transmission electron microscopy	TEM
Tungsten Inert Gas Welding	TIG
Wire-cut electrical discharge machining	WEDM
X-ray diffraction	XRD
